# Recapitulating the Pharmacological Interactions of Cetuximab with Sunitinib and Cisplatin in Head and Neck Carcinoma Cells in vitro

**DOI:** 10.1159/000527082

**Published:** 2022-10-21

**Authors:** Maria Dib, Nathanil Justian, Christian Scharf, Chia-Jung Busch, Martin Burchardt, Pedro Caetano-Pinto

**Affiliations:** ^a^Department of Ear, Nose and Throat Surgery, University Medicine Greifswald, Greifswald, Germany; ^b^Department of Urology, University Medicine Greifswald, Greifswald, Germany

**Keywords:** Drug resistance, Drug transport, Cetuximab, Cisplatin, Sunitinib

## Abstract

**Introduction:**

Cisplatin is extensively used in the treatment of head and neck carcinomas. Cetuximab combination therapy is employed in recurrent and metastatic settings. Sunitinib showed positive results in the treatment of head and neck carcinomas, both as monotherapy or in combination with cetuximab. Nonetheless, the mechanism governing these pharmacological interactions is largely unresolved. This study investigates the impact of cetuximab on the cytotoxicity of cisplatin and sunitinib using cells representative of head and neck carcinoma and the oral epithelium.

**Methods:**

The uptake and efflux activities of cells were determined using the prototypical fluorescent substrates 4-[4-[dimethylamino]styryl)-1-methyl pyridinium iodide, Hoechst 33342, and calcein-AM in the presence or absence of specific inhibitors in cells pretreated with cetuximab. The expression of key uptake and efflux drug transporters was analyzed using qPCR and immunofluorescence. Cisplatin and sunitinib cytotoxicities after cetuximab pretreatment were evaluated using the PrestoBlue viability assay.

**Results:**

Both tumor and nontumor cells showed significant active drug transport activity. Cetuximab substantially deregulated the expression of key transporters involved in drug resistance in head and neck cancer cells. Transporter expression in the nontumor cell was unaffected. Upon cetuximab pretreatment, the half maximal effective toxic concentration of cisplatin was reduced by 0.75-fold and sunitinib by 0.82-fold in cancer cells. Nontumor cells were not sensitive to cisplatin or sunitinib under the conditions tested.

**Conclusion:**

Cetuximab regulates the expression and activity of key membrane drug transporters in head and neck cancer cells, involved in drug resistance. The deregulation of the transport mechanism behind cisplatin and sunitinib uptake reverses drug resistance and enhances the cytotoxicity of both drugs.

## Introduction

Head and neck squamous cell carcinomas (HNSCCs) rank sixth among the most common cancers worldwide [1]. HNSCCs arise from the mucosal epithelium of the oral cavity, larynx, and pharynx [1]. The prevalence of HNSCCs is nowadays increasing, while the survival rates and prognosis remain poor despite the various treatment options for these malignancies [2, 3]. As treatment differs between patients, disease stages, and regional involvement, chemotherapy has long been used alone or in combination with other treatment modalities [1]. The cytostatic platinum-based drug cisplatin (CP) has long been a popular choice to treat HNSCCs. CP use is approved as monotherapy or in combination with the epidermal growth factor receptor (EGFR) monoclonal antibody cetuximab (CTX) [1, 4]. This combination therapy substantially increases survival in patients that develop CP resistance [5]. The multi-tyrosine kinase inhibitor sunitinib has been tested alone or in combination in CTX to treat HNSCCs with optimistic results [4]. However, the molecular mechanisms behind the additive effects of these therapies remain unclear.

HNSCCs originate from the mucosal epithelium and express a multitude of membrane drug transporters belonging to the ATP-binding cassette − ABC transporters and solute carrier − SLC transporters families [6]. These carriers are responsible for the accumulation and secretion of small molecules and can greatly impact the cellular toxicity of drugs [6]. CP displays limited permeability and relies on the activity of the copper transporter 1 (CTR1; SLC31A1) and organic cation transporters 1 and 2 (OCT1 and OCT2; SLC22A1 and SLC22A2) to actively enter cells from the bloodstream [7]. Its efflux is mediated through the apical multidrug extrusion protein 1 (MATE1; SLC47A1) [7]. On the other hand, sunitinib passively diffuses through the plasma membrane and acts as a substrate for the ABC transporters P-glycoprotein (Pgp; ABCB1), breast cancer resistance protein (BCRP, ABCG2), and multidrug resistance protein 4 (MRP4, ABCC4) [8]. These carriers actively efflux sunitinib from the intracellular space.

EGFR inhibition has been previously described to affect the expression and function of ABC and SLC drug transporters in epithelial cells [9]; however, its effects on the activity of HNSCCs are largely unknown. In the present study, we used two in vitro models that represent either HNSCC, the HNO97 cell line, or normal oral epithelium, the S9 cell line. Both cell lines display EGFR activity [10, 11] and were used to investigate the effects of CTX in the transport machinery of HNSCC and its impact on the cytotoxicity of CP and sunitinib.

## Material and Methods

### Cell Culture and Chemicals

HNO97 [6] and S9 [10] cells were acquired from the American Type Culture Collection(ATCC) and used as models representing HNSCC and healthy upper airway epithelium, respectively. HNO97 cells were cultured in high glucose (4.5 g/L) Dulbecco's modified eagle's medium supplemented with 10% fetal calf serum and used between passages 6 and 11. S9 cells were cultured in minimum essential medium supplemented with 10% fetal calf serum, 1% nonessential amino acids, and 2% L-glutamine and used between passages 3 and 7. Assays were performed in cells grown in microplates with about 95% confluency, usually after 2–3 days in culture. All culture mediums and supplements were acquired from PAN-Biotech (Aidenbach, Germany). CTX and CP were acquired from the University Medicine Greifswald central pharmacy. Fluorescent substrates, transport inhibitors, and sunitinib were purchased from Merck Life Sciences (Darmstadt, Germany).

### Fluorescent Functional Assays

Uptake and efflux activities were evaluated by the intracellular accumulation of fluorescent substrates. Step dilutions of substrates were performed in the presence or absence of specific inhibitors. Uptake activity was evaluated using the cationic substrate 4-[4-(dimethylamino) styryl]-1-methyl pyridinium iodide (ASP^+^, excitation: 475 nm; emission 609 nm) together with imipramine (50 µM), a potent organic cation transporter inhibitor. ASP^+^ does not passively diffuse through the plasma membrane; it requires active transport to accumulate intracellularly. Efflux activity was determined using the prototypical substrates Hoechst 33342 (excitation: 350 nm; emission 460 nm) and calcein-AM (excitation: 488 nm; emission 520 nm) for BCRP and Pgp, respectively. Both substrates passively diffuse into cells and are secreted via active transport. Hoechst 33342 binds DNA, and its intracellular retention was evaluated using the BCRP inhibitor KO143 (25 µM). In the cytoplasm, calcein-AM is metabolized into its fluorescent form − calcein − by esterase activity. Its accumulation was evaluated using the specific Pgp inhibitor PSC833 (25 µM). All functional assays were performed at 37°C using Krebs buffer. Microplates were washed twice with cold buffer after incubation (ASP^+^: 15 min; Hoeschst 33342: 30 min; calcein-AM: 45 min). Cells incubated with ASP^+^ and calcein-AM were lysed with 0.1% Triton100 in Krebs buffer. Subsequently, fluorescent intensity was acquired using a Tecan infinity 2000 microplate reader. The effects of CTX (100 μg/mL) in the uptake and efflux activities were evaluated by accessing the retention of the specific fluorescent substrates, without inhibition, after the cells were pretreated with CTX for 24 h.

### Gene Expression

The expression of relevant membrane drug transporters was determined using TaqMan^TM^ gene expression probes (Thermo Fisher, Waltham, USA), according to manufacturer specifications. The following genes were analyzed: EGFR (Hs01076090_m1), Pgp (Hs00184500_m1), BCRP Hs01053790_m1), OCT1 (Hs00427552_m1), MATE1 (Hs00217320_m1), CTR1 (Hs00977266_g1), MRP4 (Hs00988721_m1); and beta-actin (ACTB; Hs01060665_g1) was used as the reference housekeeping gene. Cells were cultured in 6-well plates until 95% confluency before treatment with 100 μg/mL CTX for 24 h; untreated cells were used as the control. Total RNA was extracted using an RNAeasy kit (Qiagen, Venlo, The Netherlands), and cDNA was generated using a high-capacity reverse-transcriptase kit (Applied Biosystems), according to manufacturer specifications.

### Immunofluorescence Characterization of Drug Transporters

Cells were grown in an 8-well slide chamber (Thermo Fisher) until confluency. Cells were washed with HBSS and fixed in 2% paraformaldehyde. For antibody staining, cells were permeabilized in HBSS with 0.1% Triton-X, for a minimum of 30 min. Cells were incubated with the primary antibodies overnight at 4°C in a solution of 0.1% Triton-X and 1% BSA (v/v) in HBSS and subsequently washed and incubated with the secondary fluorescently labeled antibody (Alexa-555 goat anti-mouse, dil:1:1000; Thermo Fisher: A-21127) for 2 h at room temperature. After the final washing step, slides were mounted and imaged in a Keyence BZ-9000 fluorescent microscope. For F-actin staining, cells were permeabilized with 2% Triton-X in HBSS; preliminary results showed poor drug transport antibody staining with 2% Triton-X, while 0.1% Triton-X showed poor phalloidin-488 (Abcam: 176763) staining in both S9 and HNO97 cells. Antibodies: BCRP, dil:1:500 (Merck; MAB4155C3); Pgp, dil:1:1000 (Novusbio; NB600-1036); OCT1, dil 1:1000 (Novusbio; NBP1-59464).

### Cell Viability Assay

Cells were pretreated with 100 μg/mL CTX for 24 h prior to CP or sunitinib exposure. CP was prepared with a maximum concentration of 2000 µM and a step dilution of 1:2. Sunitinib was prepared with a maximum concentration of 100 µM and a step dilution of 1:2. Cells were exposed to each drug for 6 h and afterward washed and kept in drug-free culture medium for an additional 24 h. After the recovery period, cells were incubated with PrestoBlue reagent (Invitrogen, Waltham, USA; 1:20 in culture medium) for 1.5 h. Immediately after incubation, the fluorescence intensity was acquired at excitation of 530 nm 590 nm emission using a Tecan infinity 2000 microplate reader (Männedorf, Switzerland).

## Data Analysis

All data were analyzed using GraphPad Prism 8 (La Jolla, California, USA). Fluorescent functional results were fitted using Michaelis-Menten kinetics to derive apparent *K*_m_ and *V*_max_ drug transport activity parameters. qPCR results were analyzed according to the 2^−ΔΔCt^ method [11], and statistically significant differences in expression, between untreated and CTX-pretreated cells, were estimated using a 2-way ANOVA. The half maximal effective concentration was determined using a nonlinear analysis of the dose-response cytotoxic effects after CP or sunitinib exposure and recovery. Immunofluorescent images were processed using the open-source software Image J [12].

## Results

### Drug Transport Activity in HNO97 and S9 Cells

**Fig. 1:**
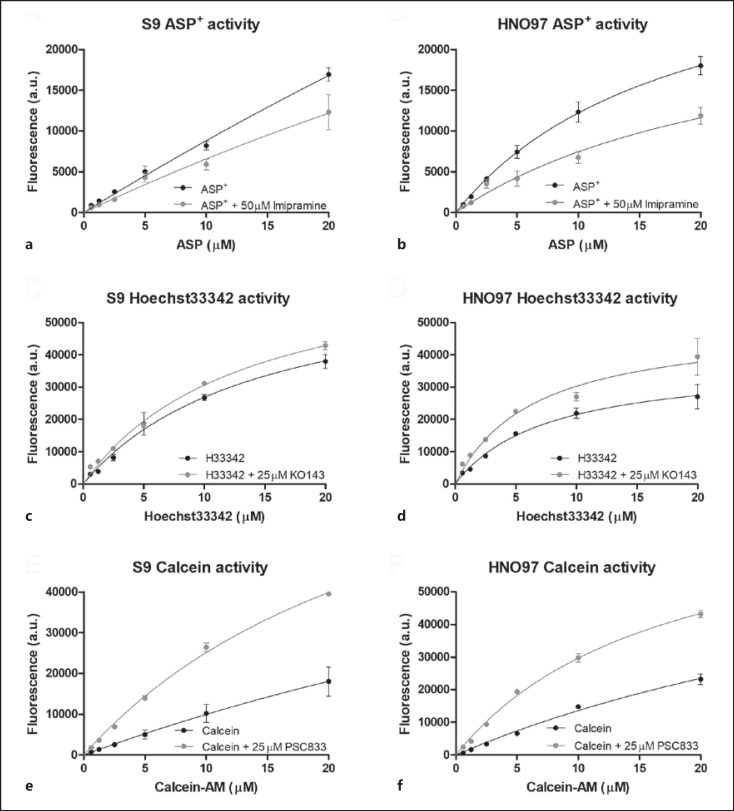
Drug transport activity in S9 and HNO97 cells. Organic cation transport activity (**a, b**; ASP^+^), BCRP activity (**c, d**; Hoechst 33342), and Pgp activity (**e, f**; calcein-AM). Functional data acquired from fluorescent assays were fitted according to Michaelis-Menten kinetics to reflect the drug transport activity of S9 and HNO97 cells. Reduced uptake of ASP^+^ in the presence of the inhibitor imipramine indicates the presence of active organic cation uptake transport. Inhibition of efflux transporters BCRP and Pgp results in increased retention of Hoechst 33342 and calcein-AM, respectively. Functional data were obtained from a minimum of two independent assays, performed in triplicates.

The accumulation of prototypical substrates ASP^+^, Hoechst 33342, and calcein were substantially inhibited, indicating that S9 and HNO97 cells have high drug transport-meditated uptake and efflux activities (Fig. 1). The apparent *K*_m_ and *V*_max_ parameters obtained from a nonlinear regression analysis of the transport activity in the presence and absence of inhibitors are described in Table 1. The little variations observed in the apparent *K*_m_ and *V*_max_ ratios between inhibited and inhibited uptake conditions (about 1–3-fold) show that transport inhibition did not change transporter affinities for the substrates while reducing their capacity. Contrary to ASP^+^ and Hoechst 33342 where *K*_m_ and *V*_max_ reflected accumulation in the cytoplasm and nucleus, respectively, in the case of calcein-AM, these parameters depend on both the enzymatic conversion of calcein-AM to calcein by esterase activity and calcein efflux, mainly by Pgp. *K*_m_ values seem to mostly reflect calcein metabolism given the differences (3-fold) observed in the presence and absence of the inhibitor. The unspecific inhibition of an MRP efflux pathway by PSC833, with lower calcein-AM affinity [13], is likely to contribute to the fluorescence accumulation observed in HNO97.

### HNO97 Shows Diminished Efflux Activity Relative to S9 Cells

**Fig. 2:**
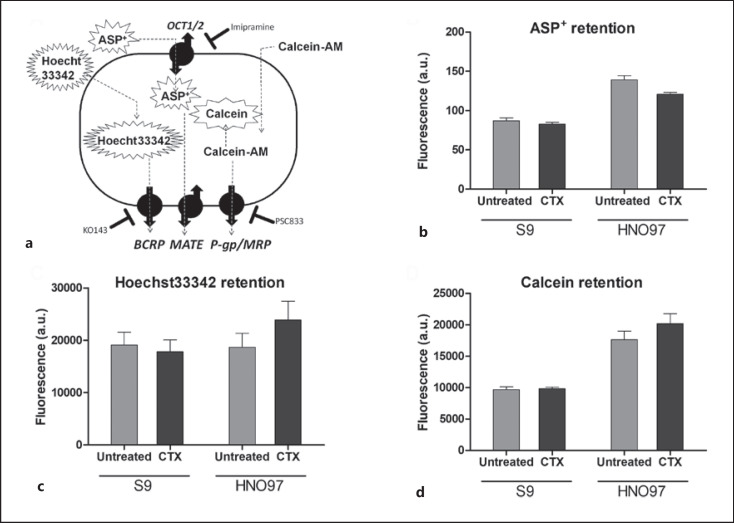
Impact of CTX in S9 and HNO97 activities. **a** Uptake and efflux routes of fluorescent substrates and respective inhibitors and membrane transporters. **b** Higher ASP^+^ intracellular fluorescence in HNO97 cells is indicative of a higher cation uptake activity, relative to S9 cells. CTX preincubation results in a slight reduction in ASP^+^ uptake in HNO97 cells. **c** Hoechst 33342 retention is similar in both cell lines, and CTX preincubation leads to a slight increase in substrate retention, indicative of reduced BCRP activity. **d** S9 Pgp activity is higher than HNO97, evident from the reduced accumulation of calcein. Fluorescent retention data were obtained from a minimum of three independent assays, performed in triplicates.

The uninhibited retention of ASP^+^ and Hoechst 33342 is similar in HNO97 and S9, revealing similar transport capacities for these substrates. Calcein retention in S9 cells is reduced by approximately 3-fold relative to HNO97 cells, an indication that the Pgp activity in S9 cells is higher. After 24 h of pretreatment with 100 μg/mL CTX, the accumulation of all substrates in S9 cells is unaffected. In HNO97 cells, ASP^+^ fluorescence is slightly decreased, and Hoechst 33342 and calcein accumulations show an increase. These observations indicate downregulation of uptake and efflux activities, respectively (Fig. 2).

### CTX Downregulates the Expression of Multiple Drug Transporters in HNO97 Cells

**Fig. 3:**
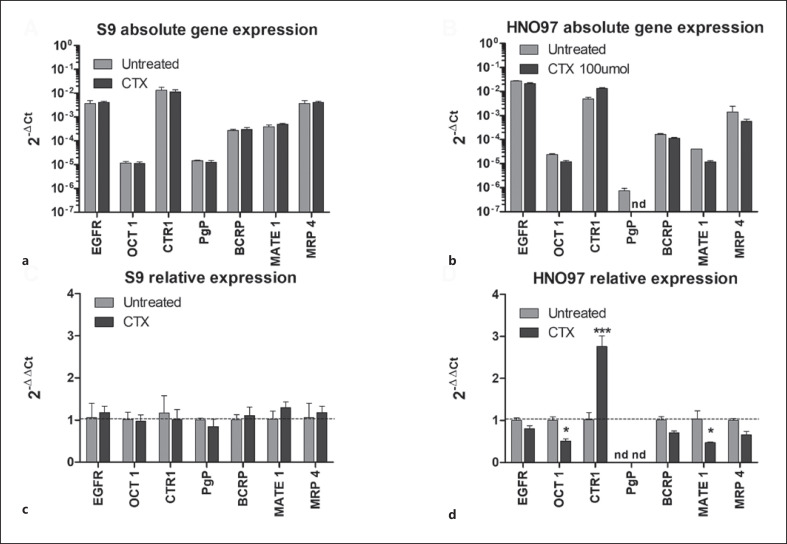
Gene expression in S9 and HNO97 cells. Absolute gene expression of key drug transporters in S9 (**a**) and HNO97 (**b**) cells and relative gene expression in S9 (**c**) and HNO97 (**d**) cells. 2^−ΔCt^ values are depicted in the log_10_ scale. Drug transporters expression was not affected by CTX pretreatment in S9 cells. In HNO97 cells after CTX pretreatment, Pgp expression was absent; the expressions of OCT1 and MATE1 were downregulated, while the expression of CTR1 was upregulated. Results represent three independent experiments. nd, not determined. *p < 0.05, ***p < 0.01.

In terms of absolute gene expression, relative to the reference gene beta-actin, both S9 and HNO97 cells display substantial expression levels of EGFR, a characteristic of epithelial cells (Fig. 3). Both cell lines show similar levels of OCT1, CTR1, BCRP, MATE1, and MRP4. The expression Pgp is substantially reduced in HNO97 in comparison to S9, and OCT2 is not expressed in either cell line. After 24 h of CTX pretreatment, the gene expression of key drug membrane transporters in S9 cells is seemingly unchanged. On the other hand, in HNO97 drug transporters, expression is mostly deregulated by CTX. The expression of efflux transporters is reduced, with the levels of BCRP downregulated by 0.7 ± 0.06-fold, MRP4 by 0.66 ± 0.12-fold, and MATE1 by 0.47 ± 0.02-fold. The expression of Pgp was completely ablated. The expression of uptake transporter OCT1 was reduced by 0.5 ± 0.08-fold, while CTR1 was the only carrier analyzed in which levels are upregulated, by 2.8 ± 0.35-fold.

### Drug Transporters Are Predominantly Expressed Intracellularly in S9 and HNO97 Cells

**Fig. 4:**
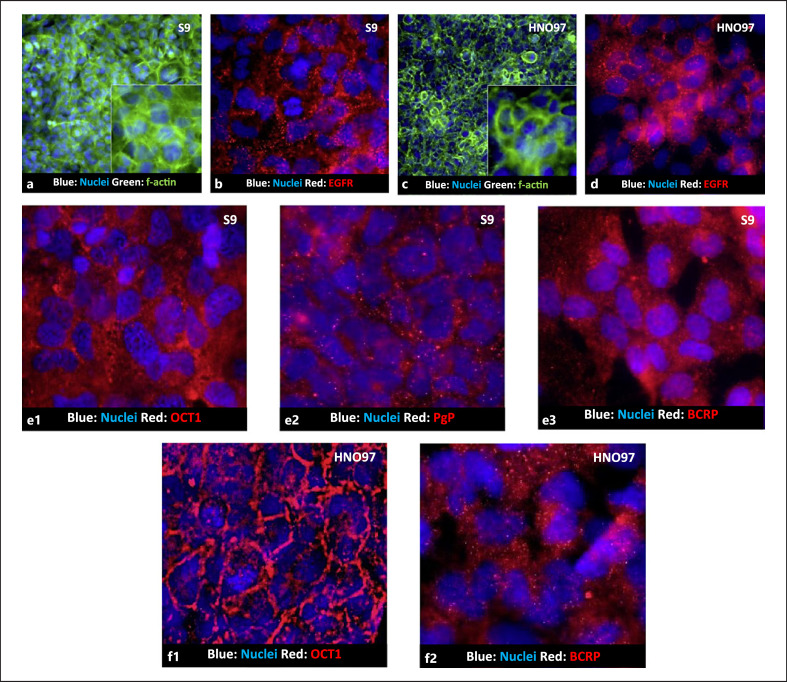
Cellular localization of drug transporters by immunofluorescence. S9 cells and HNO97 cells display a typical epithelial morphology (**a, c**) with EGFR localized in endosomes, dispersed in the cytoplasm, or the vicinity of the cell boundaries (**b, d**). In S9 cells, the expressions of OCT1 (**e1**), Pgp (**e2**), and BCRP (**e3**) are seemingly dispersed in cytoplasmic endosomes with no predominant membrane localization. In HNO97 cells, OCT1 (**f1**) is expressed in a profile corresponding to membrane localization, while BCRP (**f2**) is expressed in the cytoplasm, similar to the pattern observed in S9 cells.

Immunofluorescent characterization revealed that both S9 and HNO97 display a prototypical epithelial morphology with EGFR expressed both intracellularly in endosomes and the vicinity of the cell's boundaries (Fig. 4). In S9 cells, OCT1, BCRP, and Pgp are expressed predominantly in intracellular endosomes and not extensively found in the membrane. In HNO97 cells, the OCT1 expression pattern is consistent with membrane localization, while BCRP is also dispersed in intracellular endosomes. Given the lack of Pgp expression at the gene levels, its immunofluorescent localization in HNO97 cells was not determined.

### Selective Drug Transport Inhibition Potentiates Sunitinib Toxicity in S9 Cells

**Fig. 5:**
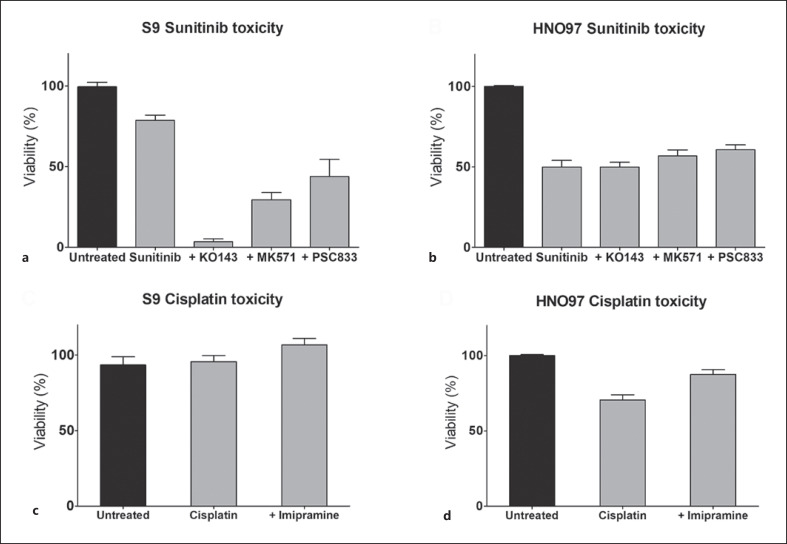
CP and sunitinib cytotoxicities in the presence of drug transport inhibitors. In S9 cells, inhibition of efflux transporter yielded a substantial increase in sunitinib cytotoxicity (**a**), while organic cation inhibition (**c**) did not affect CP toxicity. In HNO97 cells, efflux transporters inhibition did not affect sunitinib cytotoxicity (**b**). On the other hand, organic cation inhibition slightly reduced CP toxicity (**d**).

The coincubation of sunitinib with selective efflux drug transporter inhibitors resulted in enhanced sunitinib toxicity, indicating that these transporters do an active role in sunitinib clearance in S9 cells (Fig. 5). Inhibition of BCRP efflux by JO143 substantially reduced viability, to approximately 10%. To a lesser extent, MRPs inhibitor MK571 and Pgp blocker PSC833 reduced S9 viability to 30–50%, respectively. In terms of uptake, imipramine inhibition of organic cation uptake did affect CP cytotoxicity. In HNO97 cells, efflux transport inhibition did not affect sunitinib toxicity, while uptake activity inhibition resulted in a slight decrease in CP cytotoxicity. The potential toxic effects of the inhibitors used were tested, by incubating the cells with the individual inhibitors, without sunitinib or CP, for 6 h. No reduction in viability was determined (data not shown).

### CTX Enhances the Cytotoxicity of CP and Sunitinib in HNO97 Cells

**Fig. 6:**
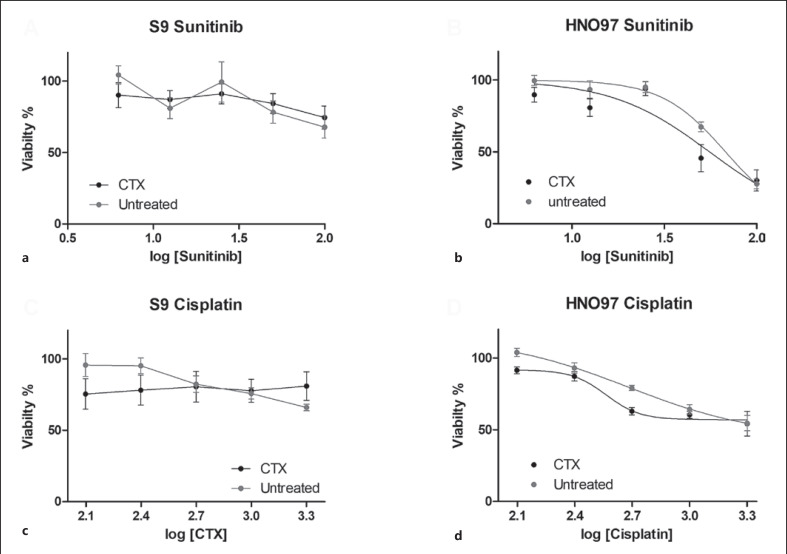
CP and sunitinib cytotoxicities in S9 and HNO97 cells. S9 cells did not show a dose-dependent response to either sunitinib (**a**) or CP (**c**) with or without CTX pretreatment. In the presence of sunitinib and CP, S9 cells showed a viability of about 70% and 80%, respectively, at the maximum concentration used. HNO97 cells had their viability reduced to 27% and 55% in the presence of sunitinib (**b**) and CP (**d**), respectively, and with or without CTX pretreatment at the maximum concentration used. EC50 values were calculated using nonlinear regression analysis. After sunitinib exposure, HNO97 cells showed a reduction in EC50 values relative to cells pretreated with CTX (67.6 µM–55.6 µM). A similar effect was observed in the EC50 values after CP exposure (no CTX: 480.7 µM, with CTX pretreatment: 365.3 µM). Cytotoxicity results were obtained from three independent experiments performed using five technical replicates. EC50, half maximal effective concentration.

In HNO97 cells, pretreatment with CTX for 24 h has an effectively enhanced CP and sunitinib toxicity, with the half maximal effective concentration of CP reduced by 0.75-fold and sunitinib by 0.82-fold (Fig. 6). In S9 cells, CP and sunitinib did not exert a dose-dependent cytotoxic effect in the presence or absence of CTX, at the concentrations used in this study.

## Discussion

By modulating EGFR activity, CTX can have far-reaching effects on the physiology of cells that positively respond to this drug, in particular those where EGFR plays a central role in regulating cellular homeostasis, growth, and survival. The beneficial tumor-suppressing effects of the combination of CTX with either CP or sunitinib are well documented. Nonetheless, the underlying molecular mechanism of these pharmacological interactions is poorly understood.

Both S9 and HNO97 recapitulate the active drug transport activity of the oral epithelium, as shown by the inhibitable uptake of prototypical fluorescent substrates, making these cells suitable to investigate the mechanisms governing the uptake and retention of chemotherapeutics. Nonetheless, immunofluorescent characterization revealed that key drug transporters are predominately localized in the cytoplasm, a phenomenon previously observed in epithelial cells in culture [14, 15]. This fact potentially precludes the drug transport activity that S9 and HNO97 in the culture conditions used. Growing both cells in conditions that enhance cell polarization (e.g., transwells) could potentially improve uptake and efflux activities.

In S9 cells, representative of a healthy oral cavity epithelium, a poor response to CP and sunitinib was observed. This is compounded by the fact that CTX does not impact the expression of key drug transporters. HNO97 cells respond to both drugs, and CTX treatment downregulates the expression of drug transporters, thus potentiating their cytotoxic effects. These findings point to the fact that drug transport regulation in S9 cells is EGFR-independent, while in HNO97 cells, this receptor is central to controlling drug transport and resistance. Efflux activity plays a key role in counteracting sunitinib toxicity in S9 cells, evident from the enhanced toxicity in the presence of efflux inhibition (Fig. 5), an effect not observed in HNO97 cells, under the same experimental conditions. OCT1 predominant membrane localization in HNO97, in contrast to its cytoplasm localization in S9, can also account for the limited CP toxicity in S9 cells. Moreover, CP cytotoxic is seemingly reduced when organic cation activity is inhibited. Another pivotal difference is the reduced expression and activity of Pgp in cancer cells relative to the noncancer cells. This feature of HNSCC can account for the sensitivity of these cancers to CP and sunitinib. Although CP is not a Pgp substrate, data have shown that Pgp overexpression is associated with CP resistance indirectly, in leukemia [12] and osteosarcoma [16] models as well as kidney injury models [14]. Studies showed that Pgp inhibits caspase activation and protects against apoptotic events, in a drug-transport independent manner [15, 17]. However, to date, the mechanisms behind this cell death-protective regulation are largely unknown. On the other hand, sunitinib is a substrate for Pgp [8]. With a reduced expression of Pgp, sunitinib is prone to accumulate intracellularly, and therefore, its toxicity is amplified.

The enhanced cytotoxicity in HNO97 promoted by CTX follows the upregulation of the CP uptake carrier CRT1 and reduction of MATE1, which facilities its cellular extrusion. CTX also reduced the expression of BCRP and MRP4, which together with Pgp mediate sunitinib efflux. The limited expression of P-gp expression in HNO97 was abolished after CTX exposure. In line with the CTX downregulation of gene expression, the accumulation of fluorescent substrates showed a decreased activity of organic cation transporters (ASP^+^ uptake) as well as BCRP and Pgp (Hoeschst 33342 and calcein efflux, respectively). The slight differences in substrate retention observed, relative to untreated cells, reflect the fact that the changes in activity are not prominent. Nonetheless, this shows a clear impact of the effects of CTX on the expression and activity of drug transporters and increased sunitinib and CP cellular cytotoxicity. By reducing drug efflux and enhancing uptake, albeit to a limited extent, CTX-mediated EGFR inhibition counteracts drug resistance in HNSCC and contributes to the beneficial effects of these combination therapies.

The impact of EGFR inhibition on drug transport function in HNSCC is an additive effect to CTX activity which leads to multiple physiological changes, notably cell cycle arrest, inhibition of proliferation, and apoptosis, which alone promotes a substantial remission of HNSCC [18]. Predicting the potential changes in the pharmacokinetic profiles of CP and sunitinib in the presence of CTX is a warranted research avenue. Given the changes in the cellular permeability to these molecules, it is important to evaluate the safety of the combination therapies in the liver and kidneys, organs with a high expression and activity of multiple drug transporters that are also sensitive to both sunitinib [19] and CP [20], respectively.

## Statement of Ethics

The present study protocol involving only in vitro experiments was reviewed, and the need for approval was waived by the Ethical Committee of the University Medicine Greifswald.

## Conflict of Interest Statement

The authors declare no conflicts of interest.

## Funding Sources

This study was supported by internal funds from the University Medicine Greifswald.

## Author Contributions

Maria Dib and Nathanil Justian contributed equally to the preparation of the present manuscript. Both authors were responsible for the experimental work, data collection and analysis, and experimental planning. Christian Scharf contributed to the experimental planning and manuscript editing. Chia-Jung Busch and Martin Burchardt provided resources and reviewed and edited the manuscript. Pedro Caetano Pinto oversaw the experimental work, manuscript preparation, and editing.

## Data Availability Statement

All the data generated or analyzed during this study are included in this article. Further inquiries can be directed to the corresponding author.

## Figures and Tables

**Table 1:** Apparent *k_m_* and V_max_ parameters derived from fluorescent functional assays in HNO97 and S9 cells

	ASP^+^		Hoechst 33342		Calcein-AM	
	*K*_m_, μM	*V*_max_, a.u.	*K*_m_, µM	*V*_max_, a.u.	*K*_m_, µM	*V*_max_, a.u.
HNO97						
Uninhibited	18.4±4.7	34,683	7.6±1.6	37,849	53.5±19.3	86,350
Inhibited	25.4±10.9	26,404	6.5±1.7	49,838	17.2±1.9	80,778
Ratio (fold)	0.7	1.3	1	0.8	3	1

S9						
Uninhibited	194.1±205	179,940	14.5±3.6	65,798	91.3±155.9	100,731
Inhibited	117.2±203	83,633	13.3±1.5	71,410	28.5±3.5	96,688
Ratio (fold)	1.7	2	1	1	3	1
